# Dose-Dependent Effects of Green Tea or Maté Extracts on Lipid and Protein Oxidation in Brine-Injected Retail-Packed Pork Chops

**DOI:** 10.3390/medicines5010011

**Published:** 2018-01-22

**Authors:** Sisse Jongberg, Mari Ann Tørngren, Leif H. Skibsted

**Affiliations:** 1 Department of Food Science, University of Copenhagen, Rolighedsvej 30, DK-1958 Frederiksberg, Denmark; ls@food.ku.dk; 2 Danish Meat Research Institute, Danish Technological Institute, Gregersensvej 9, DK-2630 Taastrup, Denmark; matn@teknologisk.dk

**Keywords:** brine injection, pork, green tea extract, maté extract, lipid oxidation, protein oxidation, modified atmosphere packaging

## Abstract

**Background:** Phenolic plant extracts are added as antioxidants in meat to prevent lipid oxidation, but depending on the concentration applied, may affect proteins either through covalent interactions or by serving as a prooxidant. **Methods:** Brine-injected pork chops prepared with green tea extract (25–160 ppm gallic acid equivalents (GAE)), or maté extract (25–160 ppm GAE) and stored (5 °C, 7 days) in high-oxygen atmosphere packaging (MAP: 80% O2 and 20% CO2) were analyzed for color changes, lipid oxidation by thiobarbituric acid reactive substances (TBARS), and protein oxidation evaluated by thiol loss and protein radical formation by electron spin resonance (ESR) spectroscopy, and compared to a control without antioxidant. **Results:** Extract of maté and green tea showed significant and comparable antioxidative effects against formation of TBARS in brine-injected pork chops for all concentrations applied compared to the control. Protein radical formation decreased significantly by addition of 25 ppm maté extract, but increased significantly by addition of 80–160 ppm green tea extract, when monitored as formation of protein radicals. Meanwhile, protein thiol groups disappeared when applying the extracts by reactions assigned to addition reactions of oxidized phenols from the extracts to protein thiols. **Conclusion:** Maté is accordingly a good source of antioxidants for protection of both lipids and proteins in brine-injected pork chops chill-stored in high-oxygen atmosphere, though the dose must be carefully selected.

## 1. Introduction

Modified atmosphere packaging and storage of meat has been attracting attention of producers, consumers, and scientists, as packaging in high oxygen concentration combined with carbon dioxide is a valuable tool for extending the microbiological shelf-life of meat. However, high-oxygen modified atmosphere packaging (MAP) may impair meat quality due to both lipid and protein oxidation [1], and these hazardous effects of MAP have been thoroughly explored in meat sold for retail, whereas meat produced and distributed for food service (canteens, nursing homes, and schools) has been given little consideration in regard to the effects of high-oxygen MAP. The food service sector is an expanding marked with a turnover of more than 5.6 billion euro per year in Denmark. Meat produced for food service must benefit from increased robustness, as the meat often is cooked and eaten in different places often undergoing reheating and longer distribution times. Especially, the tenderness and juiciness are often compromised because the meat is commonly heat-treatment to minimum 75 °C once or twice before consumption. Such robust meat products can be produced by brine-injection, which are found to stabilize the tenderness and juiciness [2]. In 2007, 28% pork and 16% beef in the US were enhanced by adding moisture through brine injection [3]. Salt and sugar added to the brine, bind water in the meat, and the meat products become more robust, gain weight, and show improved tenderness and increased juiciness after cooking [4]. 

Meat cuts sold for food service are distributed both in MAP and in vacuum, and both chilled and/or frozen. As recently reviewed by Suman et al. [5], high oxygen MAP improves meat color, but impairs the eating quality by accelerating oxidation of lipids and proteins, resulting in off-flavor formation, and less tender and juicy meat. Phenolic antioxidants extracted from herbs and spices and other botanicals, have been shown to stabilize meat color and to be efficient protectors against lipid oxidation when added to animal feed [6,7], when mixed into minced meat products [8,9], or when used in marinades for entire meat cuts [10,11]. The phenols in the plant extracts act as radical scavengers and metal chelators, inhibiting reactive oxygen species from initiating oxidation of lipids. With regards to protein oxidation, phenols have also been shown to be able to hamper oxidative protein modifications, such as in frankfurters prepared from Iberian pigs and added rosemary essential oil (150–600 ppm), where the protein carbonyl content was reduced during storage (4 °C/60 days) [12]. However, the same group found a prooxidative effect on protein carbonyls for the same experimental set-up using white pigs [13], indicating unexpected effects of phenolic antioxidants on protein oxidation, which highly depends on unknown parameters. Furthermore, studies have shown that the phenols are likely to react with the thiols or other nucleophiles in meat protein, generating protein-phenol adducts [14,15]. Any effects of formation of such adducts in fresh whole meat cuts are so far unidentified, but it has been suggested that the adduct formation may increase the protein cross-linking and polymerization, and hence increase meat toughness [14]. Most studies considering the effects of natural antioxidants applied to meat, explore the effects in minced meat of various animal origins. However, the effects of phenolic antioxidants injected into roast or chops, are less explored, even though injection-enhancement is a commonly used technology in meat production worldwide. Investigations focusing on this technology seem accordingly relevant, since phenolic antioxidants may preserve meat tenderness, a sensory quality of far more importance to whole meat cuts than for minced meats.

The aim of the present study was to investigate the dose-dependent effects of phenolic extracts from green tea and maté on the oxidative stability of MAP pork chops. Green tea (*Camellia sinensis*) is commonly used as antioxidant in various food products [16], whereas maté (*Ilex paraguariensis*) is a less utilized source of phenols. Maté consists of leaves from a South American bush and is rich in caffeic acid derivatives [17], and has successfully been used as an antioxidant added to meat or as a feed supplement for chicken and cattle protecting against lipid oxidation [7,18,19]. In the present study, the oxidative stability with regards to color, lipid, and protein was explored in MAP retail-packed pork chops cut from injection-enhanced loins containing three different levels of green tea or maté extract to compare the two sources of antioxidants including their dose-dependence on the oxidative stability of pork.

## 2. Materials and Methods

### 2.1. Plant Extracts and Chemicals

Green tea (*Camellia sinensis*) extract “Guardian Green Tea 20 M”, a commercial product with Product description PD 215033-6.0EN) was obtained from DuPont Nutrition and Biosciences ApS, Brabrand, Denmark. Maté (*Ilex paraguariensis*) extract from Centroflora, Botucatu, Sao Paulo, Brazil, was kindly provided by Daniel Cardoso at University of Sao Paulo in Sao Carlos. Details regarding extract composition and extraction method are previously published [19]. All other reagents were of analytical grade. Double-deionized water (Milipore, Bedford, MA, USA) was used throughout.

### 2.2. Total Phenolic Content in Extracts

In order to control the amount of phenols injected into the pork, the total phenolic content was determined in the maté extract by Folin Ciocalteu’s method as described by Singleton and Rossi [20]. The total phenolic content in the green tea extract was previously determined to be 23.8% [21], and this concentration was applied throughout this investigation. In brief, 100 µL 1 mg/mL maté extract dissolved to a total volume of 1500 µL double-deionized water, was left to react with 125 µL Folin−Ciocalteu phenol reagent (Sigma-Aldrich, St. Louis, MO, USA) for 8 min. Subsequently, 375 µL 20% sodium carbonate was added and the reaction mixture was left to incubate at 20 °C for 2 h. The phenol concentration was determined spectrophotometrically at 765 nm against a standard curve prepared from gallic acid. The concentrations are given in gallic acid equivalents (g/100 g dry extract; % *w*/*w*). 

### 2.3. Preparation and Storage of Injected Pork Loins

Twelve pork loins (*logissimus dorsi*) from 6 female pigs slaughtered on the same day and with similar and normal pH (~5.6) were collected from a Danish slaughterhouse and transported to the Danish Meat Research Institute (Roskilde, Denmark). The loins were divided in half to obtain 24 half loins, and four loins from the same pig were injected with either salt brine or salt brine added three different concentrations of extract. The neutral salt brine was based on water and contained 6.6% NaCl and 5.5% dextrose, which is the common composition used by the industry. The brines with extract were based on the results from the Folin Ciocalteu analysis of total phenolic content, and the neutral salt brine was added 0.10, 0.31, or 0.63% green tea extract, or 0.11, 0.34, or 0.69% maté extract, to obtain 100, 350 or 700 ppm extract, or 25, 80, or 160 ppm phenolic compounds (gallic acid equivalents, GAE) in the injected loins with an expected gain of 12% (*w*/*w*). Both left and right loins from three pigs were used for the treatment with green tea extract (*n* = 3, A, B, C), and left and right loins from three other pigs were used for the treatment with maté extract (*n* = 3, A, B, C). The loins were divided in halves, and three half loins were added the low concentration of phenolics (25 ppm), another added the medium concentration (80 ppm), and a third the high concentration (160 ppm). Additionally, one half loin from each pig were injected with salt brine, resulting in six replicates of the control without antioxidant (A–F). Samples treated with 25, 80 or 160 ppm GAE green tea extract are named GT1, GT2, GT3, respectively, and samples treated with 25, 80 or 160 ppm GAE maté extract are named M1, M2, M3, respectively. The half loins were weighted prior to injection for the determination of weight-gain. The weight-gain was further used to calculate the exact amount of phenolic compounds present in the meat:

Calculation example:

(1) Phenol content in brine: 0.10% extract in brine correspond to 0.1 g/100 mL 23.8% = 0.238 mg/mL phenol in brine
(2) Phenol in loin (2.02 kg) for a weight-gain of 229 g (mL): 229 mL 0.238 mg/mL phenol = 54.5 mg phenol
(3) Phenol (ppm) in loin: 54.5 mg phenol/2.02 kg meat = 27.0 mg phenol/kg meat = 27 ppm.

The injections were performed using a multichannel brine injector (FMG 26/52, Fomaco A/S, Køge, Denmark) with 66 punch/minute, 1.0 bar pressure, and 3.0 bar up-pressure. The injected half loins were covered in plastic bags to avoid evaporation from the surface and left to equalize overnight in the dark at 2–5 °C. Next day, the loins were dabbed with tissue and weighted for calculation of total weight-gain. The final concentration of phenolic compounds in ppm was calculated based on the total weight-gain for each loin. Every half loin was subsequently sliced in 8 pork chops of 2.0 cm and the whole loin numbered 1–16, having number 1–8 starting from the hip-end and 9–16 ending in the neck-end. The pork chops were then randomized for the various analyses, and the pork chops to be analyzed on day 1 were vacuum packed and stored cold until analysis the next day. The pork chops to be stored for day 7 were packed in modified atmosphere packaging (MAP, 80% O_2_/20% CO_2_) using a traysealer (T200, Multivac, Wolfertschwenden, Germany). Tray (K2190-53H clear/MAPET) and film (Toplex HB PET EP40 code 2600/040, oxygen permeability: 2.5 cc/m^2^/24 h) for MAP were obtained from Færch Plast (Holstebro, Denmark). No soaker pads were applied. Trays were transported to University of Copenhagen (Frederiksberg, Denmark) and stored for 7 days at 5 °C in a display cabinet with light exposure (~950 lux as measured on product surface) for 10 h daily. At the end of storage, the surface color was measured for all samples before mincing. The mince was afterwards carefully mixed, divided in smaller portions, vacuum packed and stored at −80 °C until analysis.

### 2.4. Color Analysis

Color was measured on all samples through the packaging film using a Konica Minolta Spectrophotometer CM-600d (illuminant D65, 10° standard observer, 8 mm aperture) and the corresponding Color Data Software CM-S100w SpectraMagic™ NX (Konica Minolta Sensing Inc., Osaka, Japan). CIE (Commission International de l'Éclairage) 1976 L* (lightness), a* (redness), b* (yellowness), C* (chroma), and h (hue angle) values were determined. Reflectance spectra within the visual spectrum (400–700 nm in 10 nm intervals) were measured simultaneously. Each value of the color parameters and reflectance represents an average of five measurements per sample. From L*, a*, and b* the total color difference (ΔE) was calculated [22]:

ΔE = √ (ΔL^2^ + Δa^2^ + Δb^2^) (1)

### 2.5. Lipid Oxidation by TBARS

Lipid oxidation in the pork loins was determined by TBARS analysis according to Vyncke [23] and Sørensen & Jørgensen [24] as described in Jongberg et al. [25]. TBARS were determined in the meat spectrophotometrically after reaction with 2-thiobarbituric acid at 532 nm using 600 nm as baseline. Results are expressed as 2-thiobarbituric reactive substances (TBARS) in mg MDA (malondialdehyde equivalents)/kg dry matter using a standard curve and are presented as mean ± sd (*n* = 3, but for control sample, *n* = 6).

### 2.6. Myofibrillar Protein Isolates (MPI)

Myofibrillar proteins were isolated from the pork chops according to the method described by Park et al. [26] with slight modifications as described by Koutina et al. [27]. Three replicates were prepared per sample type (*n* = 3). The myofibrillar protein isolates (MPI) were lyophilized and stored at −20 °C until analysis.

### 2.7. Protein Thiol Concentration

Protein thiol groups were determined in the MPI after derivatization with DTNB (5,5 dithiobis(2-nitrobenzoic acid, Sigma-Aldrich, St. Louis, MO, USA) [28] as previously described [29].

### 2.8. Protein Radical Detection

MPI was transferred to clear fused quartz ESR tubes (inner diameter 4 mm, wall 0.5 mm, Wilmad, Buena, NJ, USA) to reach approximately 5 cm filling of the tube. The tubes were placed in the cavity of a JEOL JES-FR30X ESR spectrometer (JEOL Ltd., Tokyo, Japan) and measured according to Gravador et al. [6]. The radical signal intensity relative to a Mn(II) standard was calculated based on the density of the sample measured as g/cm in the ESR tube [Radical intensity (Spin) = (signal area sample/signal area Mn(II))/density sample (g/cm)]. 

### 2.9. Statistical Data Analysis

Statistical analysis was performed using R^©^ version 3.4.2., The R Foundation for Statistical Computing (ISBN: 3-900051-07-0). Data were analyzed by analysis of variance using a linear model with mixed effects with the variables ‘‘Treatment’’ and ‘‘Phenol concentration’’ as fixed effects, and “End” as random effect. Where ‘‘Phenol concentration’’ was found insignificant for the statistical model, it was excluded as a variable. The significance level used was *p* < 0.05. A partial least square regression (PLSR) plot without standardization on individual observations (three per treatment) was conducted using Unscrambler (version 9.8) by applying a design matrix of injection brine, phenolic concentration and day as X-matrix, and color and oxidation parameters as Y-matrix.

## 3. Results

**Figure 1:**
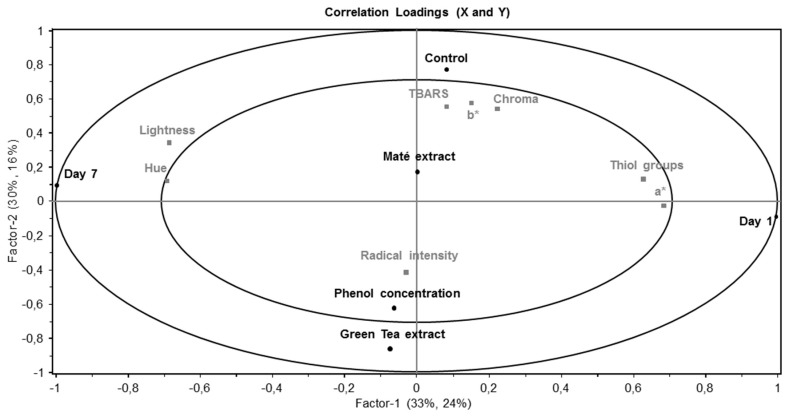
Oxidation parameters presented as a PLSR correlation loadings plot for PC1 versus PC2 of pork chops injected with three different concentrations of green tea or maté extract (25–180 ppm gallic acid equivalents), and chill stored in high-oxygen modified atmosphere packaging (MAP) for 1 or 7 days. The model was derived from treatment variables (injection brine [Control, Green tea, or maté extract], phenol concentration, and day) in the X-matrix, and color parameters and oxidation parameters in the Y-matrix. Ellipses represent *r*^2^ = 0.5 and 1.0.

A PLSR plot gave an overview of the parameters investigated in the present study (Figure 1). The correlation loadings plot shows that the variation in PC1 was primarily explained by differences between day 1 and day 7 resulting in a gradient of time going from right (day 1) to left (day 7). Day 1 associated with redness (a*) and the concentration of thiol groups, whereas day 7 associated with hue and lightness. The variation in PC2 was explained by the difference in brine with the control (salt brine) and green tea extract or the phenol concentration extending the axis in each direction. The control sample were associated with chroma, yellowness (b*) and TBARS, and green tea extract and the phenol concentration were associated with radical intensity. Maté extract was found in the middle, and closest to the control. 

### 3.1. Weight-Gain and Phenolic Content

The concentration of extract in the brine injected half loins was based on the total phenolic content determined in gallic acid equivalents by the Folin-Ciocalteu method. The phenolic content in the green tea extract was 23.8 g/100 g [21] and for the maté extract 21.7 g/100 g. The final phenol content in the brine-injected pork loins was calculated with respect to the actual weight gain in each half loin after injection and equalization (Table 1).

The calculated phenol content was found to vary more for the phenol concentration levels at 80 and 160 ppm. The variation was assigned to the variation in weight-gain between the hip- and neck-end of the loins. The gain for the hip-end was in average 8.8 ± 2.3%, which is significantly lower than for the neck-end, which was in average 13.8 ± 2.0%. From a technological point of view, this means that when injecting pork loins, the injection procedure must be adjusted according to whether it is the hip or neck part that is being subject to injection. This will ensure minimum variation in weight-gain, and hence, low variation in the extract concentration applied to each half loin. For the discussion of the results in the present study, the actual phenol concentration in each half loin is accordingly taken into consideration when evaluating the results.

### 3.2. Color Changes

The color of the injected pork loins was evaluated after 7 days of storage in MAP. Data showed that the lightness, the b-value, and chroma value were significantly different depending on the injection brines (Table 2). In the CIELAB color space, the b axis extends from blue (−b) to yellow (+b) and chroma is perceived as the strength of surface color, also defined as the brightness or colorfulness of the object. 

Results indicated that application of green tea or maté extract to the brine reduced the lightness, b* and chroma, resulting in less yellow and less bright colored pork loins. No effect of the extract concentration or interaction between injection brine and concentration on any of the color parameters were observed. Calculation of total color difference (ΔE), which is a metric for understanding how the human eye perceives color difference, resulted in ΔE > 3 for the pork chops added green tea extract. Typically, values ~2.3 corresponds to JND *(just noticeable difference*) [22]. The pork chops added the low and intermediate concentration of maté extract were <2.3 and hence, not noticeable by the human eye, whereas the high concentration of maté with ΔE = 2.7 also were within the category of JND.

### 3.3. Lipid Oxidation

**Figure 2:**
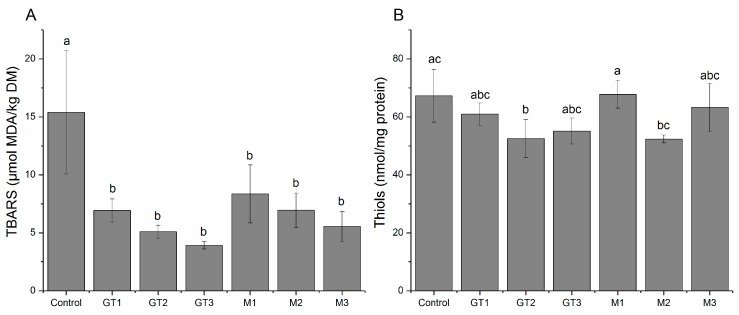
(**A**) Secondary lipid oxidation products as determined by TBARS (µmol MDA equivalents per kg dry matter) and (**B**) protein thiol concentration (nmol/mg protein) in pork chops injected with three different concentrations (denoted 1, 2, or 3 corresponding to 25, 80, and 160 ppm phenolic compounds, respectively) of green tea (GT) or maté extract (M) and chill stored in high-oxygen modified atmosphere packaging (MAP) for 7 days. Different letters (a–c) denotes significant (*p* < 0.05) difference between samples.

The secondary lipid oxidation products measured as TBARS were quantified after 1 and 7 days of storage. At day 1, no significant difference in TBARS was observed between any of the treatments (data not shown), and the average concentration was found to be 8.6 ± 1.8 µmol MDA/kg DM (mean ± sd). On the other hand, at day 7 the level of TBARS differed between treatments (*p* = 0.0018) as TBARS were found to be lower in the pork loins injected with green tea extract or with maté extract. As seen from Figure 2A, the decrease tended to be dependent on the concentration level of phenolic compounds, but no significant difference was observed between the two extracts, or between concentration levels.

Even though the brine contained equal concentrations of phenolics, based on gallic acid equivalents determined by the Folin-Ciocalteu method, the actual phenol concentrations varied between samples as shown in Table 1. For the high dose, the concentration of green tea was found to be significantly lower than the concentration of maté, which makes it difficult to compare across treatment for the high concentration level. The phenolic composition of green tea is different from the composition of maté extract, and this may also affect their antioxidative effect, as the different phenolics may have different mode of action. So, even though the reducing capacity against the Folin-Ciocalteu reagent is the same, it does not a priori provide any information about the actual mechanism by which the extracts serve as an antioxidant protecting the lipids.

**Figure 3:**
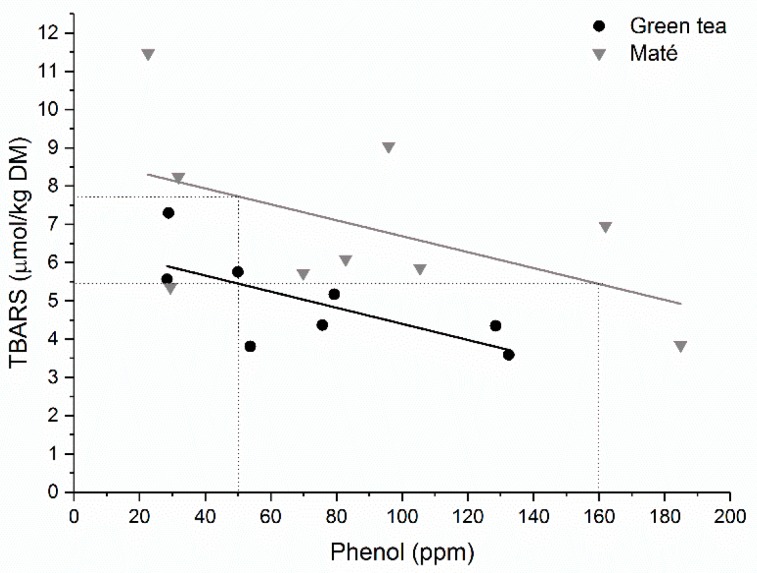
TBARS plotted as a function of phenolic content for all samples containing green tea or maté extract. Linear fit for samples added green tea extract (●): y = −0.0210x ∙ 6.50, R^2^ = 0.488, and for samples added maté extract (▼): y = −0.0208x ∙ 8.77, R^2^ = 0.272. Dashed lines indicate the concentration of phenol needed to obtain comparable TBARS levels.

The phenolic composition of green tea extract has been widely characterized and consists primarily of flavonoids, such as catechin, epicatechin, epicatechin gallate, epigallocatechin gallate, and quercetin, with lower levels of hydrocinnamic acids, caffeic, coumaric, and ferulic acid [16]. In contrast, maté extract consists primarily of caffeoyl derivatives including caffeic acid, chlorogenic acid, as well as various dicaffeoylquinic acids [17]. The maté extract used in the present study contains 58.2% chlorogenic acid and 28.4% 1,5-dicaffeoylquinic acid [19]. In meat, one of the primary oxidation initiators is the hydrogen peroxide-activated hypervalent myoglobin species, and a recent study demonstrated, that extracts from green tea and maté were equally effective in reducing the perferrylmyoglobin radical, and that the total phenolic content had more impact on the reducing capacity rather than the specific phenolic composition [30]. However, when it comes to protecting against lipid oxidation as determined by the formation of secondary lipid oxidation products, TBARS, the present study shows that green tea extract is more effective as an antioxidant as compared to maté. Figure 3 shows that both extracts protected against TBARS in a dose-dependent manner, and that the increment in effect was similar (comparable slopes) for both extracts by increasing concentrations. However, green tea is 3-fold more effective in lowering TBARS (50 ppm phenolics from green tea extract resulted in the same TBARS concentration as 160 ppm phenolics from maté extract). The correlation coefficients for the linear regressions presented in Figure 3 indicate a large variation in the pork chops added extracts. This was especially distinguished for the pork chops that had added maté extract, which makes it difficult to predict antioxidative effects in large-scale productions. This aspect has already been further investigated in our laboratory.

### 3.4. Protein Oxidation

Protein oxidation as evaluated by the loss of thiol groups were quantified in the myofibrillar fraction of the meat proteins, the MPI. No significant differences between samples were observed at day 1 (data not shown) and the average concentration was found to be 75.9 ± 10.1 nmol/mg protein (mean ± sd). At day 7, a significant loss in thiols were observed for GT2 (*p* = 0.0439), and M2 was also close to significantly reducing the thiol concentration (*p* = 0.0549), as compared to the control without antioxidant (Figure 2B). None of the other concentration levels affected the protein thiols. This suggests that green tea extract, and possibly maté extract, may decrease the thiol concentration when added to pork loins stored in high-oxygen MAP, indicating that these extracts not solely serves as antioxidants protecting against oxidation. Similar results have previously been reported [9,14,25,31], and has been explained by the reaction between the quinones, which are the oxidation products of the phenols, and the protein thiol groups [15,31]. The quinones are strong electrophilic compounds and reacts rapidly with nucleophiles, such as thiol and amino groups forming covalent protein-phenol adducts [32,33]. Addition of green tea extract (as seen for the 25–375 ppm concentration interval of phenolic compounds) to meat emulsions showed that heat treatment (70 ° for 15 min) depleted thiols in a dose-dependent manner, and it was suggested, that the excessive loss of thiols predominantly was caused by thiol-quinone adduct formation [14]. Likewise, recent studies by other groups have demonstrated that addition of chlorogenic acid [34], catechin [35], or (-)-epigallocatechin-3-gallate (EGCG) [36] to myofibrillar proteins change the physicochemical properties of the proteins, especially the gelling properties. The changes are found to be either advantageous or detrimental depending on the concentration applied, and all mentioned studies assign the changes to be caused by the formation of quinone-protein covalent interactions. Application of phenolic rich extracts has a multifaceted role in meat by being both anti- or prooxidative and playing a part in the structural development of proteins especially during heat treatment. 

**Figure 4:**
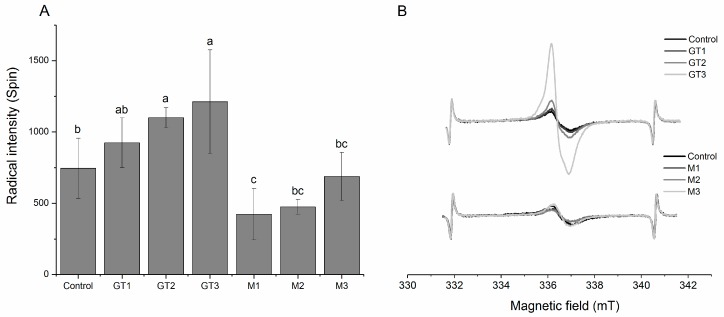
(**A**) Protein radical intensity (Spin) and (**B**) representative ESR spectra of MPI isolated from pork chops injected with three different concentrations (denoted 1, 2, or 3 corresponding to 25, 80, and 160 ppm phenolic compounds, respectively) of green tea (GT) or maté extract (M) and chill stored in high-oxygen modified atmosphere packaging (MAP) for 7 days. Different letters (a–c) denotes significant (*p* < 0.05) difference between samples.

The concentration of protein radicals was quantified using electron spin resonance (ESR) spectrometry on a relative scale expressed as radical intensity. The radical intensity did not develop significantly during the storage time of 7 days in any of the samples, therefore only day 7 results are presented in Figure 4. As seen from the figure, GT2 and GT3 were found to have significantly higher protein radical intensity, whereas M1 was found to have significantly lower protein radical intensity as compared to the control sample without extract (Figure 4B). It appeared that the green tea extract increased the protein radical intensity in a dose-dependent manner, whereas maté had no such effect, but in contrast protected against protein radical formation for the lower concentrations applied. This became evident by comparing the shape of the ESR spectra, where especially GT3 resulted in a distinctly increased signal (Figure 4B). 

An increased protein radical intensity may be caused by prooxidant effects of phenols added as plant extracts. Especially tea-polyphenols have been found to generate hydrogen peroxide upon oxidation carrying the risk of compromising the oxidative stability of food components in the presence of metal catalysts such as iron through generation of hydroxyl radicals. It has, however, also been demonstrated that food proteins may play an important role in retarding this prooxidant effects of phenolic compounds [37]. In meat, this latter property of the proteins may be essential, due to the high concentration of protein, which then may act as an endogenous antioxidative system. It seems likely, that meat proteins will scavenge radicals generated by oxidation of phenolic compounds catalyzed by transition metals. Meat proteins have high molecular weight and radicals may stabilize within the protein structure, reduce their reactivity, and accumulate in the meat as stable protein radicals. In this way, the increased protein radical intensity observed in the present study may be the result of a prooxidant effect of the green tea extract generating stable protein radicals. 

Other explanations for the elevated radical intensity should also be considered. A previous study on Bologna-type sausages added green tea extract as antioxidant showed a similar increase in radical intensity [25]. However, in that study the ESR spectra of the MPI from the control sausage versus the sausage added green tea extract were different, indicating that the radical species formed in the samples were different. It was suggested that the covalent protein-phenol adducts generated through thiol-quinone interactions, could scavenge and stabilize radicals, generating phenoxyl radicals attached to the protein, in effect increasing the radical signal intensity observed. In the present study, when comparing the shape of the ESR signal from the control sample with a sample containing green tea, no apparent difference was observed (data not shown). This indicates that different phenomena take place in the two systems, and that heat treatment might be a crucial factor for the generation of sufficient thiol-quinone adducts to act as protein-bound radical scavengers. The modest reduction in the concentration of protein thiol groups observed in the present study (Figure 4) verifies that phenols from green tea only to a lesser extent had reacted with the protein thiols. 

In contrast to the green tea extract, the maté extract decreased the radical intensity of the meat protein, and the lowest concentration of maté extract tested had the highest effect (Figure 4). A decreased radical intensity indicate that protein radicals have not been generated due to low prooxidant activity, or simply that the protein radicals have already reacted and caused damage in other targets of oxidation present in the meat.

### 3.5. Dose-Dependent Effects of Green Tea or Maté Extracts

For lipid oxidation, though not significantly, a dose-dependent effect of green tea was observed, where higher concentration of green tea extract caused lower TBARS levels. The same tendency was observed by addition of maté extract, still, without a significant decrease. For protein thiols, the level tended to decrease by at the intermediate phenol concentration, however, the dependency was only significant for green tea. For neither the TBARS nor thiol groups any significant differences were observed between green tea and maté extract added with similar phenol concentration. This was on the other hand observed for the protein radical intensity, with a significant difference between addition of the green tea or maté extract. In this case, green tea extract also showed a clear dose-dependence, elevating radical intensity when increasing extract addition. In contrast, addition of maté resulted in reduced or similar radical intensities as compared to the control samples without extract.

The difference in antioxidant mechanisms protecting against protein oxidation between the two extracts may be explained by differences in the structure of phenolic compounds. Green tea extract consists of catechins, which contains catechol structures that when oxidized are likely to form adducts with protein, even acting as prooxidants under oxidative conditions. Maté extract on the other hand, consists of caffeic acid derivatives, which may due to a higher molecular size and rigid structure will only react with nucleophiles in protein structures to a lesser degree. More studies on their individual behavior in meat and interaction with meat proteins are necessary to fully explore the potential of maté as antioxidant agents in injected meat.

## 4. Conclusions

Green tea and maté extracts reduced the formation of TBARS in brine-injected pork chops after 7 days storage in high oxygen MAP. No difference in the antioxidative protection of lipid oxidation was found between the two extracts, or between the different concentrations applied (25–160 ppm GAE). Furthermore, 25 ppm GAE maté extract reduced the formation of protein radicals as compared to the control, which indicated an antioxidative effect of maté against protein oxidation. In contrast, green tea extract was found to increase protein radical intensity especially for the higher concentrations (80–160 ppm GAE). Meanwhile, both green tea and maté extract tended to reduce protein thiol groups, and 80 ppm GAE green tea extract showed a significant loss of thiols, which may either be caused by prooxidative activity or covalent quinone-protein interactions. Maté is accordingly a good source of antioxidants for protection of both lipids and proteins in brine-injected pork, though the dose must be carefully selected.

## Figures and Tables

**Table 1:** Concentration of extract (aimed) and phenols (aimed and calculated, mean ± sd) in brine injected-pork chops.

Treatment	[Extract]_Aimed_ (~ ppm)	[Phenols]_Aimed_ (~ ppm GAE *)	[Phenols]_Calculated_ ** (ppm GAE)
Control	-	-	-
GT1	100	25	29 ± 0 ^d^
GT2	350	80	68 ± 13 ^c^
GT3	700	160	105 ± 36 ^b^
M1	100	25	28 ± 4 ^d^
M2	350	80	83 ± 11 ^b,c^
M3	700	160	150 ± 33 ^a^

* GAE = gallic acid equivalents. ** Calculated from the weight-gain of brine-injected pork loins. ^a−d^ Different letters (a–d) denotes significant (*p* < 0.05) difference between calculated concentrations of phenols.

**Table 2:** Color parameters (mean ± sd) and the total color difference (ΔE) in brine-injected pork chops.

Treatment *	Color Parameter			ΔE
	Lightness	*b*-Value	Chroma	
Control	58.78 ± 1.65 ^a^	7.34 ± 0.18 ^a,b^	7.37 ± 0.18 ^a,b^	0.0
GT1	56.45 ± 0.49 ^b,c^	4.70 ± 0.01 ^b,c^	4.71 ± 0.01 ^c^	3.1
GT2	55.96 ± 1.30 ^c^	6.06 ± 0.95 ^b^	6.13 ± 0.96 ^b,c^	3.1
GT3	56.60 ± 0.51 ^b,c^	4.83 ± 0.62 ^c^	4.86 ± 0.65 ^c^	3.3
M1	57.98 ± 1.41 ^a,b,c^	6.34 ± 0.90 ^a,b^	6.43 ± 0.97 ^a,b^	1.3
M2	58.64 ± 1.36 ^a,b^	7.20 ± 0.28 ^a^	7.26 ± 0.29 ^a^	0.2
M3	56.21 ± 0.62 ^b,c^	6.46 ± 0.76 ^a,b^	6.48 ± 0.77 ^a,b^	2.7

* Stored in high-oxygen modified atmosphere packaging (MAP) for 7 days (5 °C). ^a−c^ Different letters (a–c) denotes significant (*p* < 0.05) values within the same column.
